# ^17^O Chemical Shifts in Water

**DOI:** 10.1021/acs.jpca.5c07170

**Published:** 2025-11-24

**Authors:** Angel C. de Dios

**Affiliations:** Department of Chemistry, 8368 Georgetown University, 37th and O Streets, Northwest Washington, Washington, D.C. 20057, United States

## Abstract

How hydrogen bonding affects the ^17^O chemical shift in water is analyzed in depth in this work. It is found that the ^17^O chemical shift in water is sensitive to both hydrogen bond distance and the OH covalent bond length. The effects are greater when the water molecule is acting as an acceptor than when it is serving as a donor. Additivity is observed with multiple hydrogen bond partners. For these reasons, the hydrogen bond dependence can be summarized in terms of simple functions of the hydrogen bond distances. Successful prediction of chemical shifts in large clusters by using small model systems is demonstrated. With this outcome, it is shown that ^17^O chemical shifts of water in large systems can be estimated by simply considering the number and type of hydrogen bonds a water molecule is participating in and their respective hydrogen bond distances. Ab initio calculations have also been performed for the ^17^O shielding in water as a function of hydrogen bonding with dimethyl sulfoxide (DMSO), and with glycerol. In these cases, results likewise show that the dependence of the ^17^O chemical shift in water on hydrogen bonding is summarized by the hydrogen bond distance and whether water is acting as a donor or acceptor. Calculations suggest that in the presence of DMSO, ^17^O in water is likely deshielded while the opposite should be observed with glycerol.

## Graphical abstract

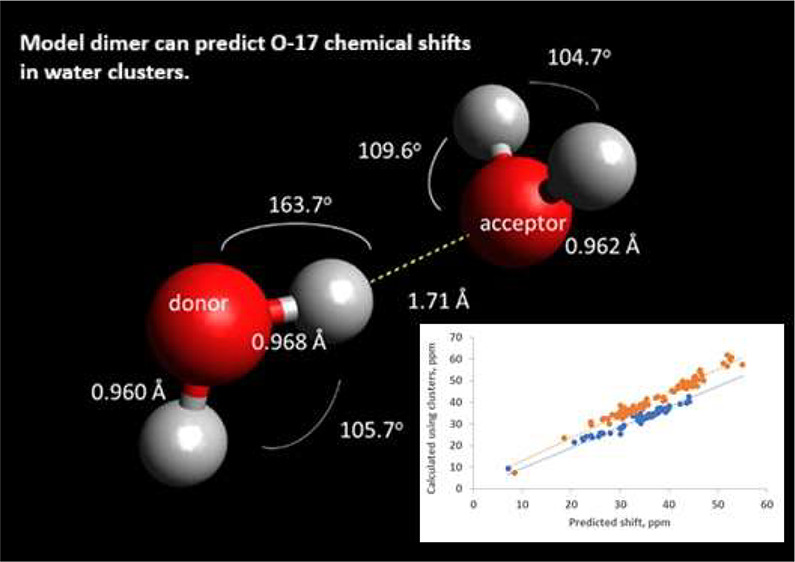

## Introduction

Water plays a vital role in the structure, stability, dynamics and function of biologically relevant molecules.^1^ Correctly reproducing the behavior of water molecules in these situations is therefore crucial in molecular dynamics simulations of biomolecular systems. Bulk properties such as dielectric constant, density, surface tension, viscosity, self-diffusion coefficient, are essential in validating models and simulations,^2^ but an atomic-level property is necessary to confirm these simulations at the molecular scale. The chemical shift in Nuclear Magnetic Resonance (NMR) spectroscopy serves as a good candidate as it is especially sensitive to short-range intermolecular interactions and molecular dynamics. The sensitivity of the ^17^O chemical shift in water to the environment has long been established.^3^ This seminal work has demonstrated the effects of ions on the ^17^O chemical shifts of water. An empirical analysis illustrates that alkali cations lead to greater shielding (negative chemical shift with respect to pure water) while anions generally cause deshielding. These opposite effects hint that the effects on the ^17^O chemical shift need to consider whether it is the hydrogen or the oxygen of water that is interacting with the ion. Moreover, correlations between the observed shifts and the size of the ions have also been noted. The effects of various solvents have likewise been measured, indicating that solvents that either provide less hydrogen bonds, or are not capable of traditional hydrogen bonding, lead to a significant shielding of ^17^O in water, unequivocally demonstrating the significance of hydrogen bonding in the ^17^O chemical shift of water. The temperature dependence has also revealed an increasing shielding with temperature, once more suggesting that hydrogen bonds cause deshielding of ^17^O in water. With these results, an estimate of about 16 ppm has been provided for the hydrogen bond contribution to the chemical shift. Covering a much wider temperature range and allowing water to go into the vapor phase, a larger estimate, 36 ppm, has been reached for this hydrogen bond contribution.^4^ This value has been further divided into two separate contributions in the case of acetic acid, namely, 12 ppm for donating a hydrogen bond and 6 ppm for accepting a hydrogen bond.^5^ Although different from water, results from acetic acid strongly imply that effects from accepting and donating a hydrogen bond need to be treated separately. Lastly, the presence of other molecules that can form hydrogen bonds with water is especially intriguing. It has been known, for instance, that NH_3_ causes greater deshielding while both acetone and trimethyl amine increases shielding. In these cases, it has been suggested that water molecules still manage to form dimers.^6^

Unfortunately, due to the relatively long NMR time scale and rapid exchange of water molecules, experimental observation has been limited in the past to obtaining only the average NMR properties of water in the ensemble.^7^ However, with huge advancements in solid state NMR, as well as computational methods, there has been tremendous progress in measuring, analyzing and interpreting ^17^O chemical shifts, as comprehensively reviewed by Wu.^8,9^ Recently, by utilizing high-magnetic fields and bulk water suppression techniques, it has been demonstrated that NMR signals from interfacial water can be observed separately.^10^ These novel ^17^O NMR experiments reveal a chemical shift for bound water that is 12 ppm more shielded than that of bulk water. The ^17^O NMR chemical shift can clearly serve as an excellent addition to experimental and computational tools that can help in the quest of understanding the molecular dynamics of water in biological systems.

To utilize ^17^O NMR chemical shifts in water, it is imperative that factors affecting this observable are understood. To achieve this, theoretical work can provide a detailed picture of how interactions and dynamics affect the ^17^O NMR chemical shift in water. Ab initio calculations of the NMR chemical shift have reached an accuracy of about 1 ppm for nuclei other than protons.^11^ Calculations of ^17^O chemical shift in water have been shown to be accurate enough to reproduce isotope shifts.^12^ The experimental ^17^O NMR chemical shift for an isolated water molecule with respect to bulk water is known,^13^ thus, calculated chemical shifts for water clusters and water molecules interacting with other molecules can be placed on the same scale as that of bulk water. In the past, it is often difficult to compare directly calculated chemical shifts against experimental values. One reason is that computations yield absolute shielding, that is, the difference between the magnetic field felt by the nucleus in a compound and the applied external field. In other words, calculated shielding values are always with respect to the bare nucleus. Experimentally determining absolute shielding values requires the measurement of spin-rotation constants. Fortunately, spin-rotation constants have already been accurately determined for water by Puzzarini and co-workers.^14^ From this work, an absolute shielding of 325.3 ppm has been measured for the ^17^O nucleus in an isolated water molecule at 300 K. Since the ^17^O absolute shielding in water at 300 K is also known, 289.2 ppm, one can deduce that the ^17^O in water at 300 K is 36.1 ppm deshielded compared to the isolated molecule.^15^ These numbers do not contain rovibrational corrections but one can perhaps assume that these corrections are almost equivalent between an isolated water molecule and bulk water such that when relative shieldings are used, these corrections largely cancel each other. While it is still impossible to calculate absolute shieldings in the liquid phase, it is straightforward to compute the shielding for an isolated molecule. Therefore, using the absolute shielding of ^17^O in an isolated water molecule, as opposed to the shielding in bulk water, as reference is consistent for theoretical work. In addition, using the isolated molecule as reference also provides the hydrogen bond contribution directly. It is important to note that referencing with respect to the isolated molecule means that the chemical shifts reported in this paper can be easily converted to chemical shifts with respect to pure water by subtracting 36.1 ppm (the difference in shielding between liquid water and an isolated water molecule).

Hydrogen bonding involves a considerable amount of orbital overlap and simplistic models such as a polarizable continuum are found to be inadequate in reproducing intermolecular NMR chemical shifts in water.^16^ Thus, it appears that supermolecule calculations are needed in calculating chemical shift effects of hydrogen bonding. An earlier attempt to extract the hydrogen bond effects on the ^17^O chemical shift in water uses a cluster of ten water molecules taken from molecular dynamics simulations.^17^ Ab initio calculations are then performed to obtain the ^17^O shielding for the central water molecule. Since the simulations also allow for changes in the internal geometry of the water molecules, large variations are seen for the calculated shielding values. The wide range of shielding values throughout the trajectory arises from rovibrational corrections. By making the water molecules less flexible, hydrogen bond effects can be isolated, which are of the right sign, but are only half of what is observed. It is expected that hydrogen bonds can alter the internal geometry of a water molecule. Variations in the internal coordinates in a molecular dynamics simulation are governed by classical force fields, thus, rovibrational corrections will differ from those obtained quantum mechanically. With these classical methods, bond lengths and angles are not at the level of accuracy required by ab initio calculations of the chemical shift. Hence, it is advisible to focus mainly on hydrogen bond parameters in predicting chemical shifts for water. Nonetheless, if classical potentials are employed for both gas and liquid phase calculations, one indeed finds that these classical rovibrational corrections are nearly the same for gas and liquid phases, as demonstrated by Malkin and co-workers.^18^ These authors have also shown that using small clusters composed of nine to 13 water molecules taken from snapshots, the average calculated shielding of the ^17^O shielding in the central water molecule already approaches the experimentally observed gas to liquid shift. More importantly, the use of density functional method (or methods with electron correlation) and large basis sets are seen as necessary in accurately calculating intermolecular chemical shifts for water.

Calculations using clusters demand a great deal of computational time. In order to obtain the shielding for each and every water molecule for every step in a simulation, a faster way of calculating shielding is needed. The water dimer is an excellent starting point as it contains the likely factors that can define the effects of hydrogen bonding on the ^17^O chemical shift in water. With the dimer, not only is the internal geometry of each water molecule considered but also the distance and orientation of hydrogen bonding. Furthermore, in the dimer, one water acts as a donor and the other serves as an acceptor, thus covering both types of hydrogen bonding a water molecule may be experiencing. The dimer is certainly an excellent starting point in unravelling how hydrogen bonding affects the ^17^O NMR chemical shift in water.

With parallel processors, chemical shifts for a system consisting of 20 water molecules with a large basis set can now be computed in about 12 h. This, however, is still limited to one static structure. For NMR chemical shifts to be useful in gauging molecular dynamics simulations, a faster way of calculating NMR shifts is needed. An ingenious way of doing this is by deriving a functional form for the NMR chemical shift in terms of intra- and intermolecular distances between the atoms in these systems, as demonstrated by Jameson et al.^19–22^ All of these can easily be provided by a smaller system like a water dimer.

Hydrogen bonding can alter the internal geometry of water especially when water acts as a donor. It is only expected that the OH covalent bond length increases with this donation. The direct effect of hydrogen bonding on the ^17^O NMR chemical shift can be dependent on both hydrogen bond distance as well as the angles. These factors can be separately examined with the use of an isolated molecule, for internal geometry changes, and with a dimer, for the hydrogen bond effects. Both isolated molecule and dimer therefore provide separately these contributions. The dependence of the chemical shift on these factors, if simple enough, can be used to generate functions that allow for a rapid computation of the chemical shift for any given situation a water molecule finds itself in a molecular dynamics simulation. However, for these functions to be valid, these should first be able to mimic results obtained using clusters at various configurations.

## Computational Details

Geometry optimization and chemical shift calculation are performed using the Gaussian program.^23^ Geometry optimization is done at the MP2 level of theory^24^ using an aug-CC-pVTZ basis set.^25,26^ NMR chemical shifts are obtained using the hybrid density functional B3LYP^27,28^ with the same basis set. Counterpoise calculations have been performed and these indicate that basis set superposition errors are less than 1 ppm and can be ignored. The calculated ^17^O shielding for an isolated water molecule at its optimized geometry is 326.14 ppm, which is used throughout this paper as reference to convert shielding into chemical shifts.

## Results and Discussion

First, the dependence of the ^17^O shielding on the geometry of the water molecule is obtained. Figure 1 displays the dependence of the ^17^O NMR absolute shielding on the HOH bond angle and OH bond length. These shielding traces are comparable to previous work.^12^ The shielding appears to be quite insensitive to changes in the HOH bond angle, changing by about 1 ppm within a range of 10°. This is fortunate, as it allows for this factor to be ignored. On the other hand, the dependence on the bond length is quite dramatic, −579.36 ppm/Å. This is the derivative of the shielding with respect to the covalent bond when both bonds are changing simultaneously at the same magnitude and direction. Therefore, if one of the O–H bonds changes by just 0.01 Å, a change of about 3 ppm in the ^17^O chemical shift is expected. This is substantial, because as mentioned previously, it is expected that the covalent bond is very likely to adjust when a water molecule donates to a hydrogen bond. Calculating the effect of hydrogen bonding on the ^17^O NMR chemical shift at various hydrogen bond distances therefore requires geometry optimization.

**Figure 1:**
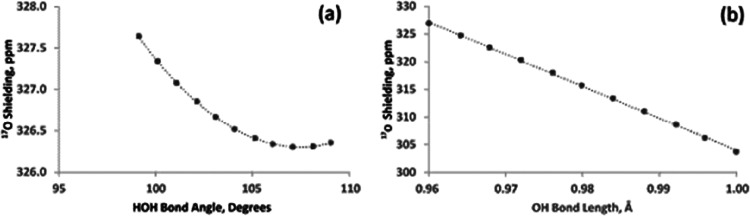
Dependence of the ^17^O absolute shielding in an isolated water molecule on (a) bond angle and (b) bond length.

The optimized structure at MP2/aug-cc-pVTZ of the water dimer is illustrated in Figure 2. The O–H covalent bond involving the H atom that is donated is longer, by almost 0.01 Å, compared to the other three covalent bonds. Since hydrogen bonding involves more than two atoms, this interaction is defined not only by distance but also orientation. In a hydrogen bond between two water molecules, one water molecule acts as a donor while the other acts as an acceptor. The effect of hydrogen bonding is expected to be different between the donor and acceptor. Fortunately, the dependence of the ^17^O chemical shift in a water dimer as a function of orientation is determined to be minimal, changing by only 1–2 ppm over a range of 30° in the HO^···^H and H^···^OH hydrogen bond angles, as shown in Figure 3.

**Figure 2:**
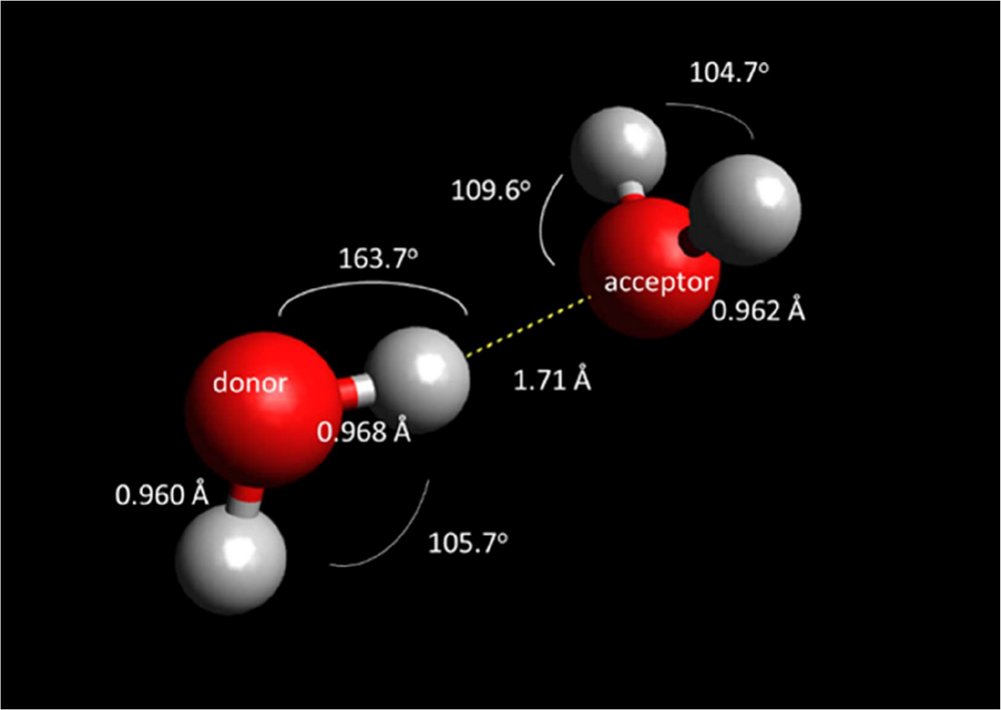
Optimized structure at MP2/aug-cc-pVTZ of the water dimer. The hydrogen bond distance HOH^···^OH_2_ is 1.71 Å, the hydrogen bond angle, O–H^···^O is 163.7°, the hydrogen bond angle, H^···^O–H is 109.6°. The internal geometry parameters of each water molecule are also shown.

**Figure 3:**
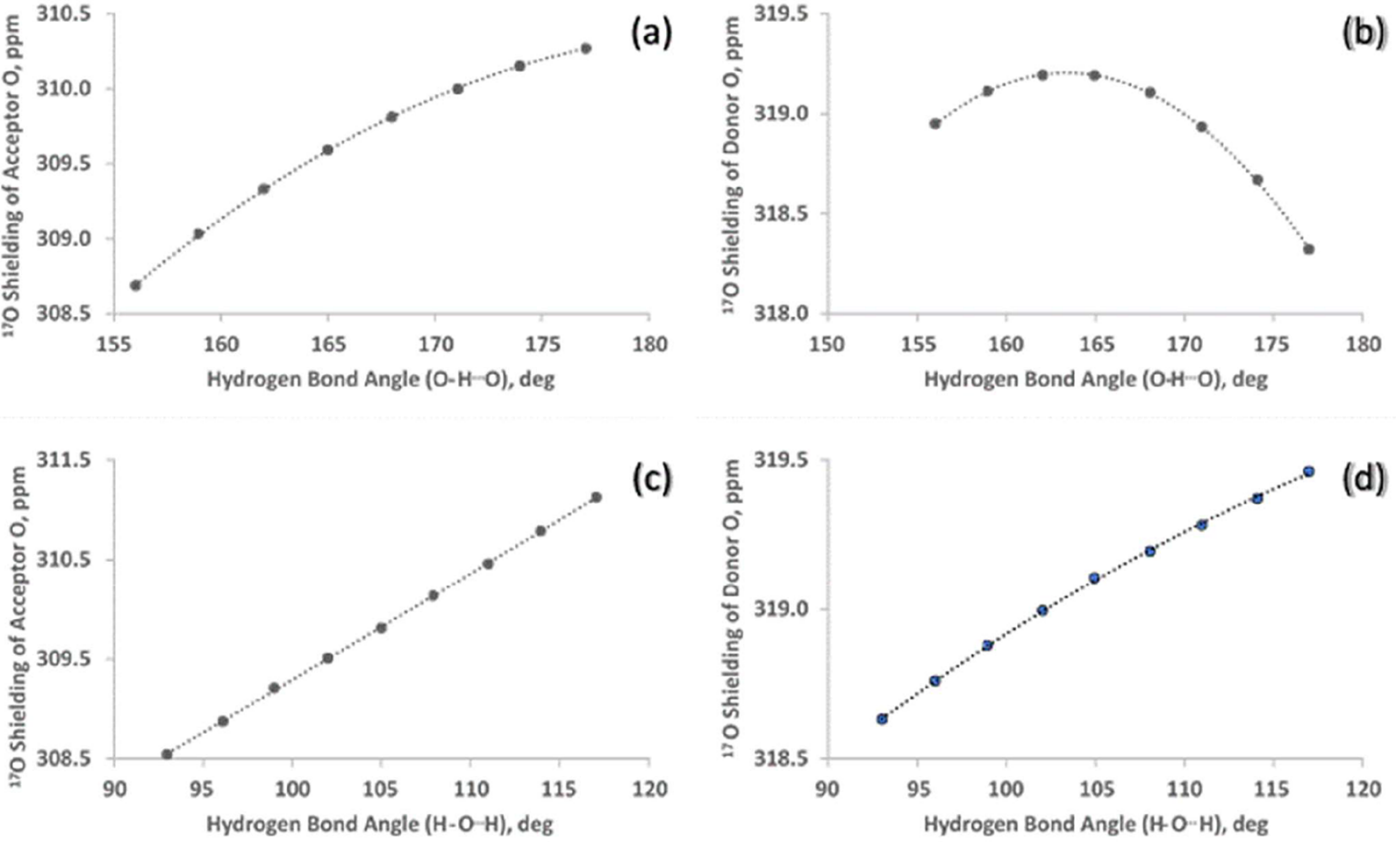
(a) The dependence of the ^17^O absolute shielding of the donor water molecule in the dimer on the OH···O hydrogen bond angle, (b) same as (a), but for the acceptor molecule, (c) the dependence of the ^17^O absolute shielding of the donor water molecule in the dimer on the H···OH hydrogen bond angle, (d), same as (c) but for the acceptor molecule.

For both acceptor and donor, the parameter that dominates the hydrogen bond effects on the ^17^O NMR chemical shift in water is the hydrogen bond distance (OH^···^OH), the distance between H (donor) and O (acceptor), as displayed in Figure 4. A (1/*r* ^4^) functional form is used as this goes to zero at infinite separation. Choosing the isolated molecule as reference makes this functional form appropriate. The range of hydrogen bond distances covered by these points is 1.7–2.0 Å. The fit is good (*R* ^2^ = 0.99 for both cases). The slope for the acceptor is 137.08 ppm Å^4^ and for the donor, 60.98 ppm Å^4^. The (1/*r* ^4^) functional form is interesting as it relates to what may be expected for an intermolecular interaction that is a combination of dipole–dipole (1/*r* ^3^) as well as orbital overlap (1/*r* ^6^). It is noteworthy to point out that the ^17^O NMR chemical shift of the acceptor is more sensitive to the hydrogen bond distance (about twice as much) compared to the donor site. This is not surprising because as an acceptor, the O atom, whose chemical shift is being examined, lies closer to the other water molecule. Moreover, the nonbonding orbitals on oxygen dramatically change when the O atom begins to accept a hydrogen bond. These results are obtained with geometry optimization of both water molecules while fixing the hydrogen bond distance. It therefore contains contributions from changes in the covalent bond length. Without geometry optimization, the corresponding slopes for acceptor and donor are 120 and 40 ppm Å^4^, respectively. These values are smaller especially for the donor as the OH covalent bond becomes longer with decreasing hydrogen bond distance. This is an additional deshielding effect, the same in sign as the effect of hydrogen bonding. The model dimer consists of one water molecule donating while the other is accepting the hydrogen bond. It is anticipated that this model works perfectly only for the cases in which water is participating in only one hydrogen bond. When a water molecule is already accepting a hydrogen bond, its covalent OH bond is more susceptible to increase when it simultaneously donates to another.

**Figure 4:**
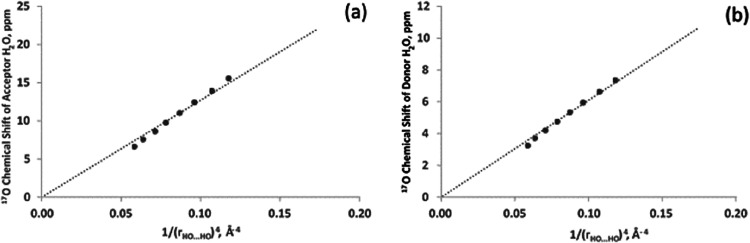
Dependence of the ^17^O chemical shift (referenced to an isolated water molecule) in a water dimer on the hydrogen bond distance (a) for the acceptor site and (b) for the donor site.

This deviation is expected to be greater when water participates in two, three, or four hydrogen bonds. (Water can formally have as many as four hydrogen bonds per molecule.). To investigate what happens with additional hydrogen bonding, models involving a trimer, a tetramer, and a pentamer, are necessary. Using a model trimer, for instance, one can calculate the effect of either donating two hydrogen bonds (2D), accepting two hydrogen bonds (2A), or accepting and donating one hydrogen bond (1A1D). Since these computations only aim to examine the effect of additional hydrogen bonds, these can be performed without geometry optimization. Based on the optimized dimer, an OH bond length of 0.962 Å is used for a non-hydrogen bonding H and 0.968 Å is used for a hydrogen bonded H.

Results of calculations for these models with a distance of 1.7 Å for all hydrogen bonds are presented in Table 1. The last column in this table is a simple sum of 16.5 ppm for each acceptance (A) and 7.4 ppm for each donation (D), the respective values determined from the dimer. For example, ab initio calculations of the ^17^O chemical shift in a trimer model for a central water molecule that is donating one hydrogen bond and accepting one (1A1D) yields 23.4 ppm. Assuming additivity, that is, 1A1D = 1A + 1D produces 23.9 ppm. This additivity and the dominance of the hydrogen bond distance suggest that the dimer can reproduce the values for various scenarios of types and number of hydrogen bonds. This result is quite intriguing as a hydrogen bond is often regarded as an intermediate between a covalent bond and weak intermolecular forces. For the NMR chemical shift, it would seem that an accurate determination requires orbital descriptions of a water dimer, which are necessary for covalent bond formation, but for a combination of hydrogen bond effects on the chemical shift, we have discovered that additivity can be assumed, which is often the case for weak intermolecular forces.

**I tbl1:** Additivity of Hydrogen Bond Effects on ^17^O Chemical Shifts in Water at 1.70 Å from Dimer, Trimer, Tetramer and Pentamer Models

number and type of hydrogen bond	cluster (ppm)	predicted using additivity (ppm)
1A	16.5	
1D	7.4	
1A1D	23.4	23.9
2A	30.8	33.0
2D	16.4	14.8
2A1D	41.4	40.4
1A2D	34.9	31.3
2A2D	49.9	47.8

To validate the use of these donor and acceptor chemical shift functions, a sufficient number of distinct ^17^O sites in hydrogen bonded water molecules are needed. For this purpose, the water clusters^29^ provided by molecular dynamics simulations followed by ab initio geometry optimization are utilized. These clusters are shown in Figure 5.

**Figure 5:**
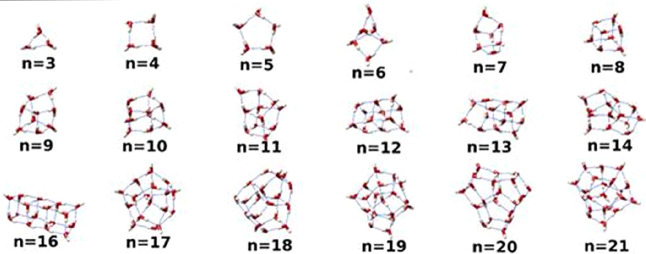
Minimum energy structures of the (H_2_O)_*n*_, *n* = 3–21 clusters (ref 29) that were used in the ab initio calculations of ^17^O shielding.

The clusters employed in the present work range from three to twenty-one water molecules (excluding the cluster of 15 H_2_O molecule). Since these structures are optimized at a high enough level of theory, the O–H covalent bond lengths, to which the chemical shift is particularly sensitive, can be utilized with confidence. The need for accurate bond lengths cannot be overstated since chemical shifts are especially sensitive to bond lengths. It is therefore fortunate that these optimized geometries are obtained at a relatively high level of theory and with a sufficiently large basis set. This is the main reason why the cluster with 15 H_2_O molecules is not included since this has not been optimized at the MP2 level. With this consideration, it therefore becomes apparent that this set of clusters allows for the determination of the adjustment factors necessary to utilize the functions from the model dimer in predicting chemical shifts in clusters. Analyzing these results should yield a scaling factor necessary to mitigate the fact that the model dimer has only one hydrogen bond. The ^17^O NMR chemical shift for the water molecules in the lowest energy structure for each cluster is calculated using the same basis set. The average ^17^O chemical shift of water in each of the clusters is shown in Figure 6a.

**Figure 6:**
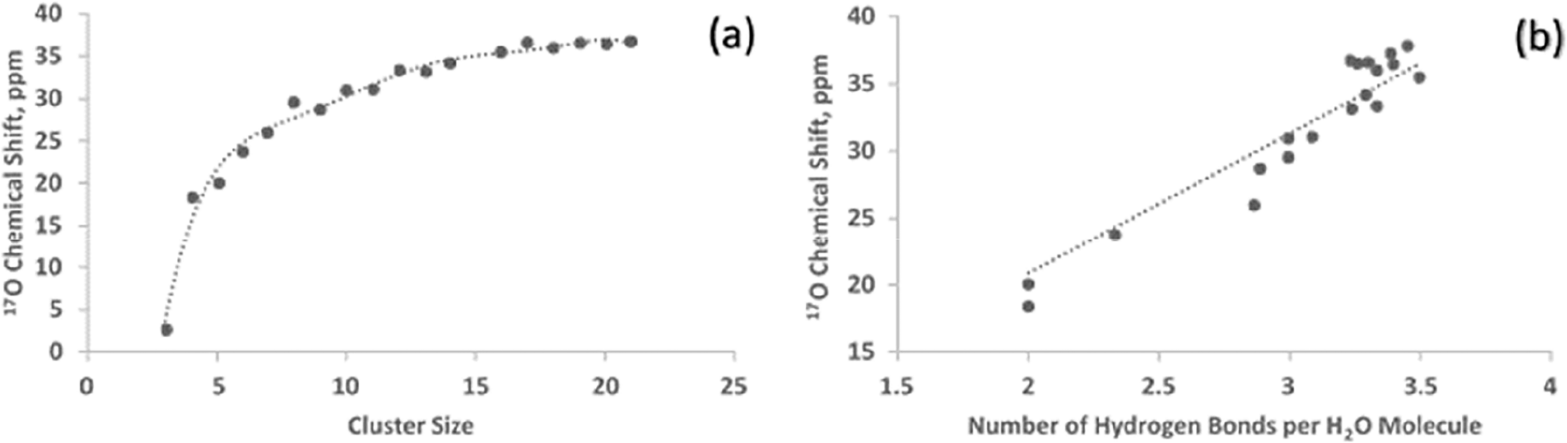
(a) The average ^17^O chemical shift (referenced to an isolated water molecule) of water as a function of cluster size and (b) The average ^17^O chemical shift (referenced to an isolated water molecule) of water as a function of the number of hydrogen bond.

It is encouraging to see that as the cluster size becomes bigger, the chemical shift approaches 36 ppm, in agreement with the observed gas to liquid ^17^O chemical shift of water.^13,15^ How the chemical shift qualitatively depends on the number of hydrogen bonds per water molecule is depicted in Figure 6b. The linear fit, also suggesting additivity, provides a value of 10 ppm per hydrogen bond, which translates to roughly 35 ppm when there are 3.5 hydrogen bonds per water molecule in liquid water. There is considerable scatter in Figure 6b. This is anticipated since there is a range of hydrogen bond distances in these sites, thus, the chemical shift cannot be predicted by simply using the number of hydrogen bonds. Nevertheless, the value of 10 ppm per hydrogen bond is already qualitatively useful. Next, we can test whether the ^17^O NMR chemical shifts for the clusters can be predicted by applying the functions of the model water dimer. All that is necessary are the hydrogen bond distances for each of the ^17^O sites in a cluster, assuming this is the only parameter required to reproduce the chemical shifts calculated using clusters. Of course, it should also be noted that it is important to distinguish donating from accepting molecules. It is also necessary to limit the hydrogen bond to the first shell. Thus, an upper limit of 2.5 Å for the OH^···^O distance (hydrogen bond distance) is used to identify hydrogen bonds. If agreement between cluster calculations and those predicted by the model dimer is reached, then the dimer chemical shift equations can be employed in determining ^17^O NMR chemical shift for water in any hydrogen bonding situation. The results are shown in Figure 7.

**Figure 7:**
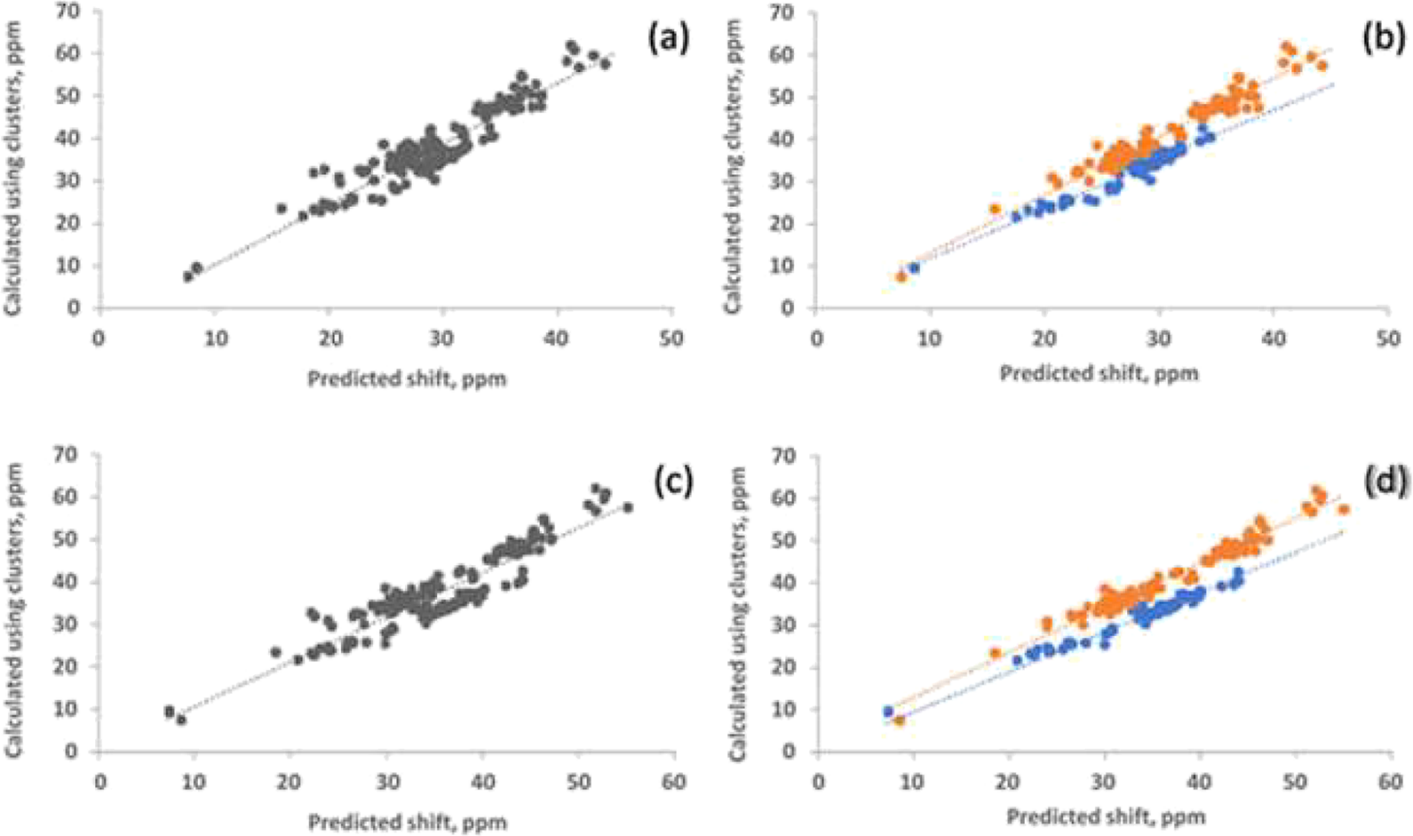
(a) Ab initio ^17^O chemical shifts for water using the cluster are plotted against values predicted using the water dimer model. The linear fit has a slope of 1.43 with an intercept of −4.05 and an *R* ^2^ of 0.87. (b) same as (a), but sites (shown in orange) donating two hydrogen bonds, no dangling OH, are separated from those (shown in blue) that are donating only one hydrogen bond, dangling OH. The fit for the no dangling sites has a slope of 1.38 with an intercept of −0.78 and an *R* ^2^ of 0.96. The fit for the dangling sites has a slope of 1.20 with an intercept of −0.64 and an *R* ^2^ of 0.95. (c) Ab initio ^17^O chemical shifts for water clusters are plotted against values obtained using the water dimer model with corrections for OH covalent bond length. The linear fit has a slope of 1.05 with an intercept of 0.08 and an *R* ^2^ of 0.84. (d) same as (c), but sites (shown in orange) donating two hydrogen bonds (no dangling OH), are separated from those (shown in blue) that are donating only one hydrogen bond (dangling OH). The fit for the no dangling sites has a slope of 1.12 with no intercept and an *R* ^2^ of 0.97. The fit for the dangling sites has a slope of 0.95 with no intercept and an *R* ^2^ of 0.96.

It is evident that the model dimer is capable of reproducing ^17^O NMR chemical shifts in this wide range of environments. One of the reasons why this happens is the approximate additivity of hydrogen bond effects on the ^17^O shifts in water as suggested by Table 1. As mentioned previously, an expected pitfall of the model dimer is its inability to predict correctly the contribution from changes in the covalent OH bonds. Although the geometry of the dimer is optimized at each hydrogen bond distance, the dimer has only one hydrogen bond. With additional hydrogen bonds, the change in the covalent bond length is different from what is derived using just a dimer. When a water molecule is accepting a hydrogen bond at the same time it is donating, the lengthening of the OH covalent bond is expected to be bigger. As shown in Figure 1b, an increase in the OH bond length leads to deshielding, or a larger chemical shift. Predicted values from the dimer, which do not fully consider the contributions from an increasing OH bond length, therefore underestimate the chemical shifts in the clusters. This is demonstrated by a slope that is greater than unity when one compares the cluster values against those predicted by the model dimer. This discrepancy becomes larger when the water molecule is donating two hydrogen bonds. Hence, it is expected that the difference depends on whether the water molecule is donating one hydrogen bond (has a dangling O–H) or donating two hydrogen bonds (no dangling). This becomes visible when the sites are separated into these two cases as illustrated in Figure 7b. Adding contributions due to bond length changes brings the predicted values closer to the cluster values as demonstrated in Figure 7c,d. The slope of the best-fit line in Figure 7c is now closer to unity, 1.05, and there is no need for an intercept. In Figure 7d, dangling sites are separated from no dangling sites, with the dangling sites having a slope of 0.95 and the latter having a slope of 1.12. Clearly, by considering changes in the covalent bonds, the predicted values using the model dimer can be brought closer to the ab initio values obtained using the clusters, lending confidence to the notion that the discrepancy seen between cluster and predicted values arises mainly from changes in the OH covalent bond.

Agreement between cluster calculations and those obtained by applying the model dimer results indicates that the effects of hydrogen bonding on ^17^O chemical shifts have been correctly identified. These results are extraordinary since only interatomic distances are required, specifically, the distance between an accepting O and a H atom that is being donated. The effects of hydrogen bonding on the ^17^O chemical shift in water seem no different from the scenario of two rare gas atoms interacting with each other.^30^ Since the correlation between cluster and predicted values is already satisfactory, one can simply use the equations for the best-fit lines in Figure 6b to predict the ^17^O chemical shift of water in any given situation. With the equations below, one can calculate the ^17^O chemical shift for any given water molecule requiring only hydrogen bond distances, and considering if the water is donating one (dangling) or two (no dangling) hydrogen bonds:

$$\delta_{\left(Oi\right)}\;\mathrm{in}\;H_{2}O\;\mathrm{clusters}\;\mathrm{predicted}\;\mathrm{for}\;O_{i}\;\mathrm{with}\;\mathrm{no}\;\mathrm{dangling}\;O-H=1.38\{\Sigma_{\mathrm{dw}}137.08[r({O_{i}}^{\cdot \cdot \cdot }H-O_{\mathrm{dw}}){]}^{-4}+\Sigma_{\mathrm{aw}}60.98[r({O_{\mathrm{aw}}}^{\cdot \cdot \cdot }H-O_{i}){]}^{-4}\}-0.78$$ 1

$$\delta_{\left(Oi\right)}\;\mathrm{in}\;H_{2}O\;\mathrm{clusters}\;\mathrm{predicted}\;\mathrm{for}\;O_{i}\;\mathrm{with}\;\mathrm{dangling}\;O-H=1.20\{\Sigma_{\mathrm{dw}}137.08[r({O_{i}}^{\cdot \cdot \cdot }H-O_{\mathrm{dw}}){]}^{-4}+\Sigma_{\mathrm{aw}}60.98[r({O_{\mathrm{aw}}}^{\cdot \cdot \cdot }H-O_{i}){]}^{-4}\}-0.64$$ 2

where dw corresponds to a donor and aw to an acceptor.

Employing eqs 1 and 2 allows for the rapid calculation of ^17^O NMR chemical shift for water thereby paving the way to determining an average value for the entire trajectory in a molecular dynamics simulation of bulk water.

In order to extend this model to systems of biological interest, the effects of intermolecular interactions on the ^17^O NMR chemical shift in water by neighbor molecules other than water need to be examined. Since these interactions are similar to those among water molecules, it is expected that for these cases, small model clusters can help predict chemical shifts in much larger clusters. An excellent candidate for this extension is dimethyl sulfoxide (DMSO). DMSO is capable of accepting a hydrogen bond and it has methyl groups that can serve as a model for CH^···^O interactions. Fortunately, the interaction between DMSO and a water molecule has already been studied extensively.^31^ From this previous work, several optimized structures for DMSO·H_2_O are already available. The three structures shown in Figure 8 illustrate a variety of interactions that are possible between DMSO and water. In Conf1, the water molecule is donating a hydrogen bond to the O atom of DMSO while accepting two nonconventional hydrogen bonds (O^···^HC), one from each methyl group. In Conf2, in addition to the donation of a hydrogen bond to the O atom of DMSO, the water molecule is accepting only one O^···^HC hydrogen bond. Lastly, in Conf3, the water molecule is only interacting with the methyl groups of DMSO. This last structure therefore provides the opportunity of examining only the effects of a methyl group to the ^17^O NMR chemical shift of water. A simple model that can be used for this interaction is a water molecule interacting with a methane molecule. This model is constructed by replacing the OS-CH3 group in Conf3 with just a hydrogen thereby transforming DMSO into a methane molecule. In this model, only one of the four H atoms in methane lies close to the O atom of water. The ^17^O NMR chemical shift can therefore be derived as a function of this distance and the results are shown in Figure 9a. No geometry optimization is performed in this case since scaling can be determined later on when comparing against the results derived from Conf3 of DMSO·H_2_O. Unlike the results for the water dimer, the shielding function in CH_4_·H_2_O (Figure 9a) is closer to an *r* ^–6^ dependence, with a slope of 1694.2 ppm/Å^6^. Interestingly, this is the form of the function that is apparent for species interacting via London forces where the shielding change is mainly due to orbital overlap.^30^

**Figure 8:**
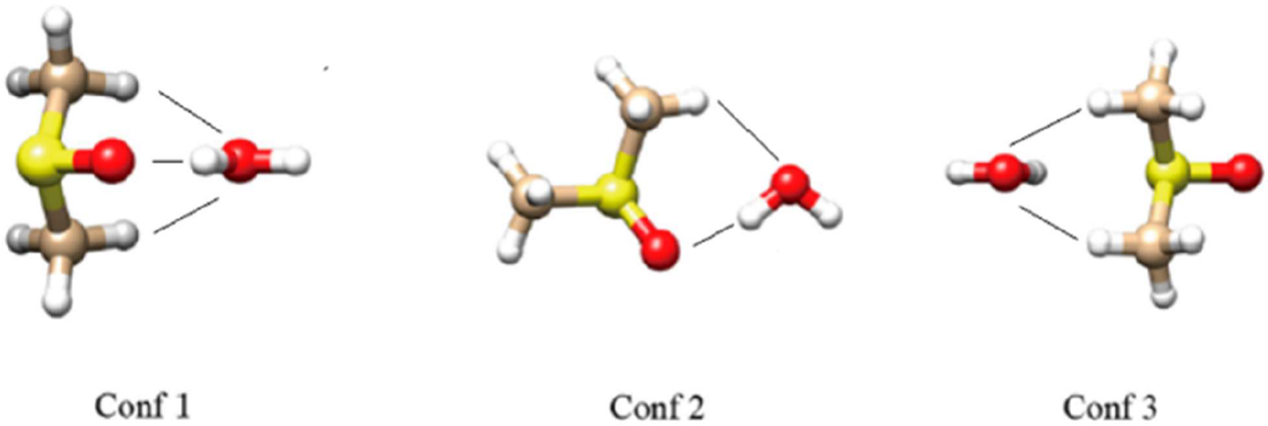
Optimized structures of DMSO·H2O used in this work. In Conf1, water donates a hydrogen bond to O=S and accepts from two H–C’s. In Conf2, water donates a hydrogen to O=S and accepts from one H–C. In Conf3, water accepts from two H–C’s.

**Figure 9:**
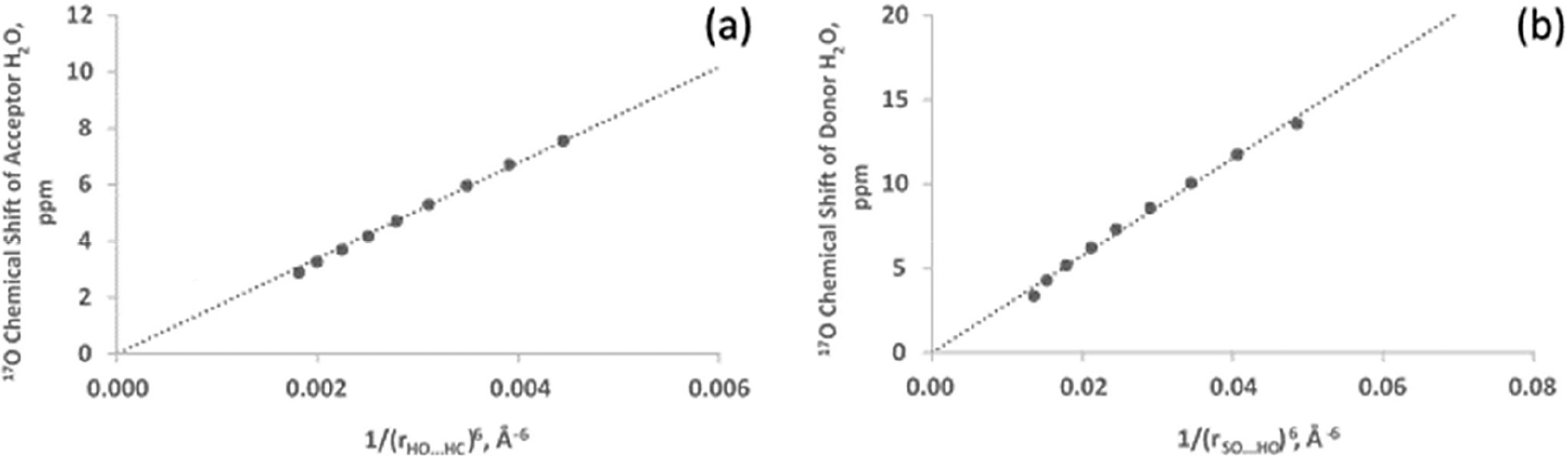
(a) The dependence of the ^17^O chemical shift of water in CH_4_·H_2_O on the HO^···^HC distance, (b) The dependence of the ^17^O chemical shift in DMSO:H_2_O as a function of SO^···^HO distance.

The three structures exhibit different types and numbers of hydrogen bond interactions. One can begin with Conf3, since this structure involves only O^···^HC interactions. After deciphering the contributions from O^···^HC, one can then proceed to Conf2 to extract specifically the OH^···^OS contribution. With both O^···^HC and OH^···^OS contributions determined; one can use Conf1 as a test case. Using the model in Figure 9a to predict the ^17^O chemical shift for Conf3 at various distances between the water molecule and DMSO yields a scaling factor of 1.13. Using this scaled dependence of the ^17^O chemical shift on CH^···^OH distance and additional shielding calculations involving Conf2 at various distances, where both SO^···^HO and CH^···^HO interactions are present, one can extract the dependence of the ^17^O chemical shift of water as a function of the SO^···^HO distance alone. This is shown in Figure 9b, which also suggests an *r* ^–6^ dependence. The slope of the best-fit line is 286.99 ppm/Å^6^. Compared to the water dimer, the effect is about twice as big, which should not be surprising since the O atom in DMSO, via population analysis in the ab initio calculation, is carrying a negative charge. Finally, to obtain a working function for DMSO·H_2_O, these functions are tested against Conf1 at various distances, in which a scaling factor of 0.94 is obtained. Thus, the following equation describes the effects of DMSO on the ^17^O chemical shift of water when water donates one hydrogen bond to DMSO and interacts with both methyl groups symmetrically as in Conf1, with the first term arising from hydrogen bonding and the second term coming from the water-methyl interaction.

$$\delta_{\left(O\right)}\;\mathrm{in}\;H_{2}O\;\mathrm{predicted}\;\mathrm{with}\;\mathrm{DMSO}\cdot H_{2}O\;\mathrm{in}\;\mathrm{Conf}1\;\mathrm{at}\;\mathrm{various}\;\mathrm{distances}=270.49[r(O-H^{\cdot \cdot \cdot }O-S){]}^{-6}+2(1799.10)[r(H-O^{\cdot \cdot \cdot }H-C){]}^{-6}$$ 3

One can test the combination of the water dimer model and the above DMSO·H_2_O equations using hydrates of DMSO where neutron diffraction structures are available.^32^ These are shown in Figure 10. The nearest neighbors to the water molecules of interest in both dihydrate and trihydrate are obtained by symmetry operations provided by the space group of the crystal. For the cluster calculations, it is necessary to optimize the hydrogen positions in the neutron diffraction structure before performing chemical shift calculations on the clusters.^33^

**Figure 10:**
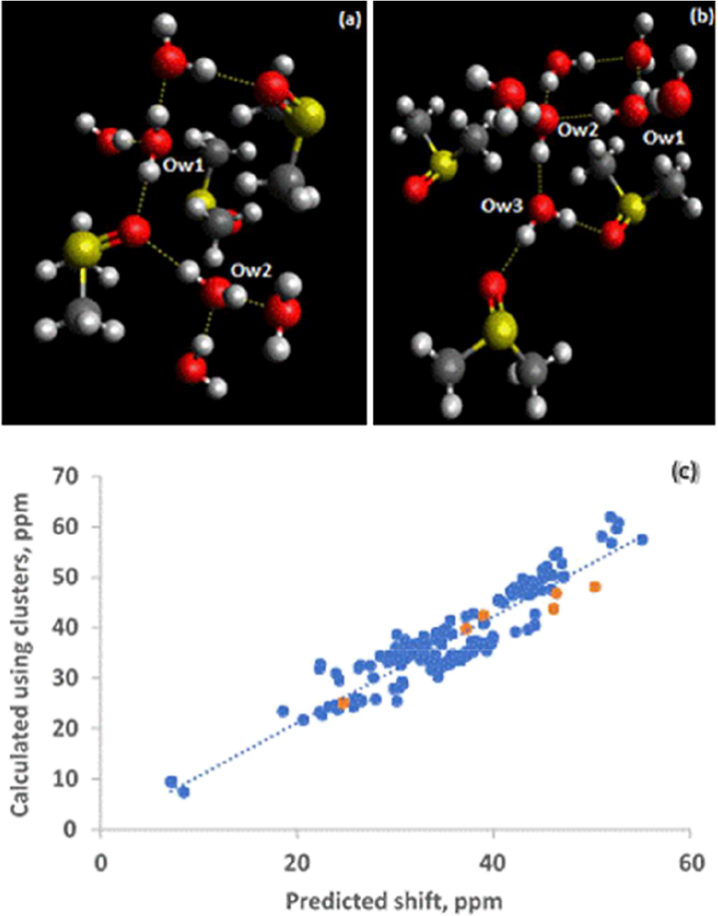
(a) The neutron diffraction structure of the dihydrate with the water molecules (Ow1 and Ow2) and its closest neighbors, (b) The neutron diffraction structure of the trihydrate with the water molecules (Ow1 Ow2 and Ow3) and (c) Agreement between cluster and predicted chemical shifts is shown by adding the points (in orange) for the dihydrate and trihydrate to the plot for water clusters shown in Figure 6c.

The chemical shifts for the hydrates of DMSO are also useful in a qualitative assessment of molecular dynamics simulations of water-DMSO solutions. For example, in a simulation of a DMSO-water solution at a mole fraction of 0.25 DMSO, the relative populations for water molecules are reported.^34^ Using what is known now regarding the effects of DMSO and hydrogen bonding between water molecules on the ^17^O chemical shifts of water, it is predicted that the average ^17^O chemical shift for H_2_O in this 0.25 mole fraction DMSO·H_2_O solution is about 1.2 ppm deshielded from that of pure H_2_O. Adding DMSO to water is expected to yield a positive chemical shift from that of bulk water simply because chemical shifts for the water molecules in the dihydrate and the trihydrate, on average, are respectively, 43.3 and 44.7 ppm more deshielded than an isolated water molecule. This is to be compared with bulk water which is only about 35 ppm deshielded.

Finally, another system that may be of application to how chemical shifts in water behave in biological systems consists of water molecules interacting with glycerol. Glycerol is capable of both donating and accepting hydrogen bonds and at the same time, also has protons attached to carbon which can likewise interact with water. Since the −OH groups of glycerol are quite similar to that of water, one may assume that the same functional forms of chemical shift equations for water–water, eqs 1 and 2, are applicable. For the H–C contributions, one may use the second term in eq 3;

$$H-C\;\mathrm{contribution}\;\mathrm{to}\;\delta_{\left(O\right)}\;\mathrm{in}\;H_{2}O=(1799.10)[r(H-O^{\cdot \cdot \cdot }H-C){]}^{-6}$$ 4

To test this hypothesis, glycerol–water clusters derived from simulations and ab initio geometry optimization can be employed.^35^ One of these structures is shown in Figure 11. Not all of the structures are used in this paper: Only those that have water molecules hydrogen bonded to glycerol and other water molecules. Furthermore, the ^17^O chemical shifts of the OH groups in glycerol are not included in the analysis. Lastly, since these optimized geometries are not obtained using the same level as the water clusters analyzed previously, the prediction may not perform as well on these glycerol–water clusters. Similar to the treatment applied to water clusters, chemical shifts computed using the glycerol–water supermolecules can be compared to what is predicted by applying eqs 1, 2, and 4 that make use of the relevant (O^..^H) interatomic distances observed in the cluster. Initial comparison suggests that predicted values overestimate the chemical shifts in the glycerol–water clusters.

**Figure 11:**
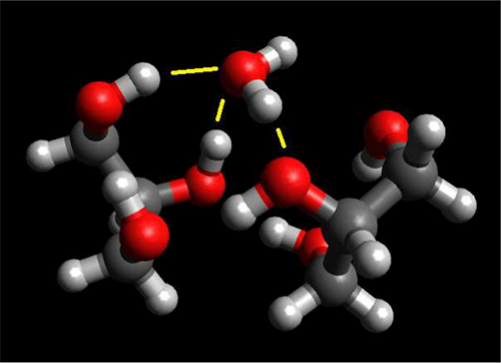
One of the glycerol–water clusters employed in this paper. This cluster is composed of one water and two glycerol molecules. The water molecule (top center) is accepting two hydrogen bonds from a glycerol molecule while donating a hydrogen bond to the other glycerol molecule. This cluster also illustrates a water molecule with a dangling O–H bond.

Figure 12 shows a better agreement between cluster and predicted shifts if a scaling factor of 0.9 is applied to eqs 1 and 2 when a glycerol molecule is the hydrogen bond partner of water. The scaling factor of 0.9 automatically reduces the effect of the hydrogen bond when glycerol is either the donor or acceptor. This alone predicts that water in glycerol–water mixtures is expected to be more shielded than bulk water, opposite to that in DMSO-water solutions.

**Figure 12:**
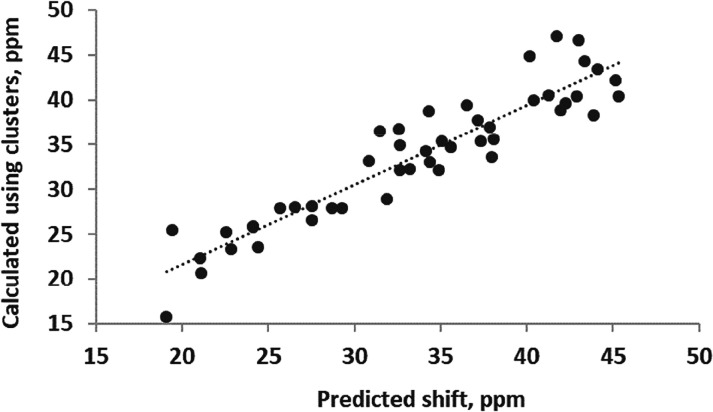
Ab initio ^17^O chemical shifts for water in glycerol–water clusters are plotted against values predicted using eqs 1 and 2, scaled by 0.9 if glycerol is the hydrogen bond partner and eq 3 for CH^..^O contributions. The slope of the best fit line is 1.00 (no intercept) and *R* ^2^ = 0.86.

Lastly, since the principal components of the chemical shift tensors are also obtained in these calculations, it may be useful to note the following observed trends. The calculated ^17^O chemical shift tensor span for the isolated water molecule is 61.4 ppm. The changes in the tensor upon accepting and donating a hydrogen bond are opposite in sign. Donating one hydrogen bond increases the span to 82.6 ppm while accepting one hydrogen bond decreases the span to 45.0 ppm. Simultaneously donating and accepting hydrogen bonds leads to a span of 63.6 ppm. These effects are clearly not additive. A water molecule participating in four hydrogen bonds, two donating and two accepting, has a span of 43.1 ppm. Water molecules participating in three hydrogen bonds have tensor spans ranging from 40.9 to 53.3 ppm. These values agree with those observed for water in various hydrates in the solid state.^36,37^

## Conclusions

We have discovered that the change in the ^17^O shielding in water upon formation of hydrogen bond can be described by a function of the form *r* ^–4^ where r is the hydrogen bond length for both an acceptor and a donor. The sign is the same for both, with the acceptor having nearly twice the effect compared to the donor. By employing a dimer model describing the ^17^O chemical shift as a function of hydrogen bond distance, equations describing the dependence on interatomic distances between the atoms of water and the atoms of its nearest neighbor can be derived and these effects turn out to be additive when tested against a large number of ^17^O sites in water clusters of optimized geometry. The comparison against cluster values affords a way of circumventing the nonadditivity suggested in a previous work.^38^ It is true that the hydrogen bond effects on the ^17^O chemical shift in water come from a combination of direct and indirect factors. The indirect factor comes mainly from the lengthening of the OH covalent bond when the water molecule is donating to a hydrogen bond. This discrepancy is negligible for the acceptor chemical shift but becomes significant for the donor. The lengthening of the O–H bond correlates with a decreasing hydrogen bond distance. These two effects therefore go in the same direction. Therefore, the factors contained in eqs 1 (nondangling) and 2 (dangling) essentially correct for this additional internal geometry effect. And as seen in Figure 6d, when bond lengths are accounted for, the slope between cluster and predicted values is near unity. Using these equations, it has likewise been adequately demonstrated that ^17^O chemical shifts of water in water, in DMSO-water, and in glycerol–water clusters can be predicted. With this approach, it is now possible to incorporate chemical shift calculations into molecular dynamics simulations as the use of these equations do not increase substantially the required computational resources. Since differences are already observed with DMSO and glycerol, it is expected that the equations presented here describing the hydrogen bond dependence of the ^17^O chemical shift in water may not be applicable to other functional groups such as C=O and N–H. Models specific to these groups are perhaps necessary. Nevertheless, the ^17^O chemical shift in water is an excellent addition to the experimental values that can be utilized to validate results of simulations. And last, it is strongly advisible to use defining parameters for a hydrogen bond similar to those provided by Luzar and Chandler;^39^ distance and angular criteria, namely, *r*_(OH···OH)_ < 2.5 Å and O–H^···^O hydrogen bond angle >150°. In this manner, only the first solvation shell around a water molecule is considered.

## References

[1] Levy Y., Onuchic J. N. (2004). Water and Proteins: A Love-Hate Relationship. Proc. Natl. Acad. Sci. U. S. A..

[2] Kadaoluwa
Pathirannahalage S. P., Meftahi N., Elbourne A., Weiss A. C. G., McConville C. F., Padua A., Winkler D. A., Costa Gomes M., Greaves T. L., Le T. C., Besford Q. A., Christofferson A. J. (2021). Systematic Comparison of the Structural and Dynamic
Properties of Commonly Used Water Models for Molecular Dynamics Simulations. J. Chem. Inf. Model..

[3] Luz Z., Yagil G. (1966). Water 17O Nuclear Magnetic
Resonance Shift in Aqueous Solutions of
1:1 Electrolytes. J. Phys. Chem..

[4] Florin A. E., Alei Jr. (1967). 17O NMR Shifts in
H217O Liquid and Vapor. J.
Chem. Phys..

[5] Reuben J. (1969). Hydrogen-Bonding
Effects on Oxygen-17 Chemical Shifts. J. Am.
Chem. Soc..

[6] Florin A. E., Alei M. Jr. (1969). The Nature of Water
Species in Water-Ammonia Solutions
as Inferred from Proton and Oxygen-17 Nuclear Magnetic Resonance Observations. J. Phys. Chem..

[7] Mathur-De
Vré R. (1980). The NMR Studies of Water in Biological Systems. Prog. Biophys. Mol. Biol..

[8] Wu G. (2008). Solid-state
17O NMR studies of organic and biological molecules. Prog. NMR Spectrosc..

[9] Wu G. (2019). 17O NMR studies
of organic and biological molecules in aqueous solution and in the
solid state. Prog. NMR Spectrosc..

[10] Zhang R., Cross T. A., Peng X., Fu R. (2022). Surprising Rigidity
of Functionally Important Water Molecules Buried in the Lipid Headgroup
Region. J. Am. Chem. Soc..

[11] de
Dios A. C., Jameson C. J. (2015). Recent Advances in Theoretical and
Physical Aspects of NMR Chemical Shifts. Kimika.

[12] Wigglesworth D., Raynes W. T., Sauer S. P. A, Oddershede J. (1999). Calculated
Nuclear Shielding Surfaces in the Water Molecule; Prediction and Analysis
of σ­(O), σ­(H) and σ (D) in Water Isotopomers. Mol. Phys..

[13] Makulski W., Wilczek M., Jackowski K. (2018). 17O and 1H
NMR Spectral Parameters
in Isolated Water Molecules. Phys. Chem. Chem.
Phys..

[14] Puzzarini C., Cazzoli G., Harding M. E., Vázquez J., Gauss J. (2009). J.
Chem. Phys..

[15] Wasylishen R. E., Bryce D. L. (2002). A Revised Experimental
Absolute Magnetic Shielding
Scale for Oxygen. J. Chem. Phys..

[16] Klein R. A., Mennucci B., Tomasi J. (2004). Ab Initio
Calculations of17O NMR-Chemical
Shifts for Water. The Limits of PCM Theory and the Role of Hydrogen-Bond
Geometry and Cooperativity. J. Phys. Chem. A.

[17] Chesnut D.
B., Rusiloski B. E. (1994). A Study
of NMR Chemical Shielding in Water Clusters
Derived from Molecular Dynamics Simulations. J. Mol. Struct.: THEOCHEM.

[18] Malkin V. G., Malkina O. L., Steinebrunner G., Huber H. (1996). Solvent Effect on the
NMR Chemical Shieldings in Water Calculated by a Combination of Molecular
Dynamics and Density Functional Theory. Chem.
- Eur. J..

[19] Yuan H., Murad S., Jameson C. J., Olson J. D. (2007). Molecular Dynamics
Simulations of Xe Chemical Shifts and Solubility in N-Alkanes. J. Phys. Chem. C.

[20] Jameson C. J., Sears D. N., Murad S. (2004). Molecular
Dynamics Averaging of Xe
Chemical Shifts in Liquids. J. Chem. Phys..

[21] D
Stueber D., Jameson C. J. (2004). The Chemical Shifts of Xe in the
Cages of Clathrate Hydrate Structures I and II. J. Chem. Phys..

[22] Sears D. N., Jameson C. J. (2003). Theoretical Calculations of the Xe Chemical Shifts
in Cryptophane Cages. J. Chem. Phys..

[23] Frisch, M. J. ; Trucks, G. W. ; Schlegel, H. B. ; Scuseria, G. E. ; Robb, M. A. ; Cheeseman, J. R. ; Scalmani, G. ; Barone, V. ; Petersson, G. A. ; Nakatsuji, H. ; Li, X. ; Caricato, M. ; Marenich, A. V. ; Bloino, J. ; Janesko, B. G. ; Gomperts, R. ; Mennucci, B. ; Hratchian, H. P. ; Ortiz, J. V. ; Izmaylov, A. F. ; Sonnenberg, J. L. ; Williams-Young, D. ; Ding, F. ; Lipparini, F. ; Egidi, F. ; Goings, J. ; Peng, B. ; Petrone, A. ; Henderson, T. ; Ranasinghe, D. ; Zakrzewski, V. G. ; Gao, J. ; Rega, N. ; Zheng, G. ; Liang, W. ; Hada, M. ; Ehara, M. ; Toyota, K. ; Fukuda, R. ; Hasegawa, J. ; Ishida, M. ; Nakajima, T. ; Honda, Y. ; Kitao, O. ; Nakai, H. ; Vreven, T. ; Throssell, K. ; Montgomery, Jr., J. A. ; Peralta, J. E. ; Ogliaro, F. ; Bearpark, M. J. ; Heyd, J. J. ; Brothers, E. N. ; Kudin, K. N. ; Staroverov, V. N. ; Keith, T. A. ; Kobayashi, R. ; Normand, J. ; Raghavachari, K. ; Rendell, A. P. ; Burant, J. C. ; Iyengar, S. S. ; Tomasi, J. ; Cossi, M. ; Millam, J. M. ; Klene, M. ; Adamo, C. ; Cammi, R. ; Ochterski, J. W. ; Martin, R. L. ; Morokuma, K. ; Farkas, O. ; Foresman, J. B. ; Fox, D. J. Gaussian 16, Revision A.03; Gaussian, Inc.: Wallingford CT, 2016.

[24] Frisch M. J., Head-Gordon M., Pople J. A. (1990). A Direct MP2 Gradient Method. Chem. Phys. Lett..

[25] Kendall R. A., Dunning T. H., Harrison R. J. (1992). Electron
Affinities of the First-Row
Atoms Revisited. Systematic Basis Sets and Wave Functions. J. Chem. Phys..

[26] Woon D. E., Dunning T. H. (1993). Gaussian Basis Sets
for Use in Correlated Molecular
Calculations. III. The Atoms Aluminum through Argon. J. Chem. Phys..

[27] Becke A. D. (1993). Density-Functional
Thermochemistry. III. The Role of Exact Exchange. J. Chem. Phys..

[28] Lee C., Yang W., Parr R. G. (1988). Development
of the Colle-Salvetti
Correlation-Energy Formula into a Functional of the Electron Density. Phys. Rev. B.

[29] Rakshit A., Bandyopadhyay P., Heindel J. P., Xantheas S. S. (2019). Atlas of Putative
Minima and Low-Lying Energy Networks of Water Clusters N = 3–25. J. Chem. Phys..

[30] Jameson C. J., de Dios A. C. (1992). Ab initio Calculations of the Intermolecular Chemical
Shift in Nuclear Magnetic Resonance in the Gas Phase and for Adsorbed
Species. J. Chem. Phys..

[31] Lv D., Evangelisti L., Maris A., Song W., Salvitti G., Melandri S. (2022). Characterizing
the Interactions of Dimethyl Sulfoxide
with Water: A Rotational Spectroscopy Study. J. Phys. Chem. A.

[32] Fortes A. D., Ponsonby J., Kirichek O., García-Sakai V. (2020). On the Crystal
Structures and Phase Transitions of Hydrates in the Binary Dimethyl
Sulfoxide–Water System. Acta Crystallogr..

[33] Liu F., Phung C. G., Alderman D. W., Grant D. M. (1996). Carbon-13 Chemical
Shift Tensors in Methyl Glycosides, Comparing Diffraction and Optimized
Structures with Single-Crystal NMR. J. Am. Chem.
Soc..

[34] Vishnyakov A., Lyubartsev A. P., Laaksonen A. (2001). Molecular Dynamics Simulations of
Dimethyl Sulfoxide and Dimethyl Sulfoxide–Water Mixture. J. Phys. Chem. A.

[35] Lu W., Mackie C. J., Xu B., Head-Gordon M., Ahmed M. (2022). A Computational and Experimental
View of Hydrogen Bonding in Glycerol
Water Clusters. J. Phys. Chem. A.

[36] Michaelis V. K., Keeler E. G., Ong T.-C., Craigen K. N., Penzel S., John, Kroeker S., Griffin R. G. (2015). Structural Insights into Bound Water in Crystalline
Amino Acids: Experimental and Theoretical 17O NMR. J. Phys. Chem. B.

[37] Nour S., Widdifield C. M., Libor Kobera, Kevin, Errulat D., Terskikh V. V., Bryce D. L. (2016). Oxygen-17
NMR Spectroscopy of Water Molecules in Solid Hydrates. Can. J. Chem..

[38] Pennanen T. S., Lantto P., Hakala M., Vaara J. (2011). Nuclear Magnetic Resonance
Parameters in Water Dimer. Theor. Chem. Acc..

[39] Luzar A., Chandler D. (1993). Structure and Hydrogen Bond Dynamics
of Water-Dimethyl
Sulfoxide Mixtures by Computer Simulations. J. Chem. Phys..

